# Supplementary material for: Time Trends in Ischemic Heart Disease Mortality Attributable to PM2.5 Exposure in Southeastern China from 1990 to 2019: An Age-Period-Cohort Analysis
Source: Int J Environ Res Public Health. 2023 Jan 5;20(2):973. doi: 10.3390/ijerph20020973 (PMC9859070; doi:10.3390/ijerph20020973)
Supplement: Supplementary file 1 [file ijerph-20-00973-s001.zip › ijerph-2063516-supplementary.pdf]

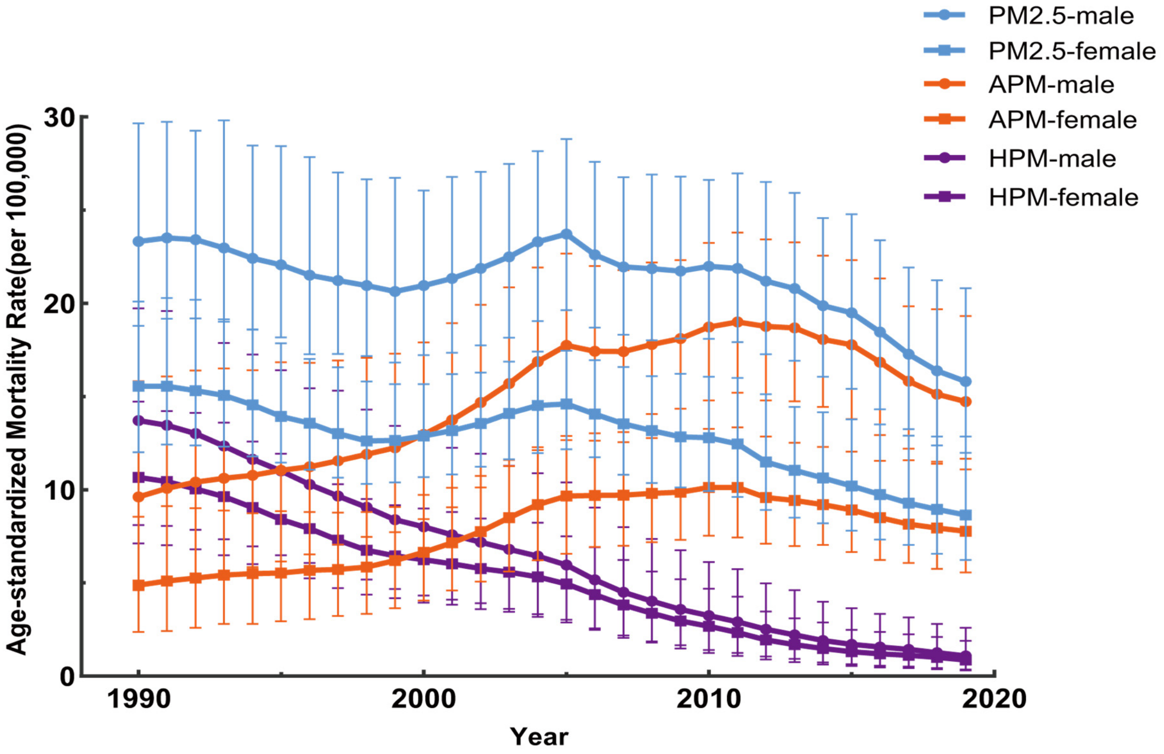

Figure S1. The temporal trends in the ASMR of IHD attributable to $PM_{2.5}$ (APM and HPM) in Jiangsu, 1990 to 2019. The dots and error bars denote ASMR and their corresponding 95% CI.

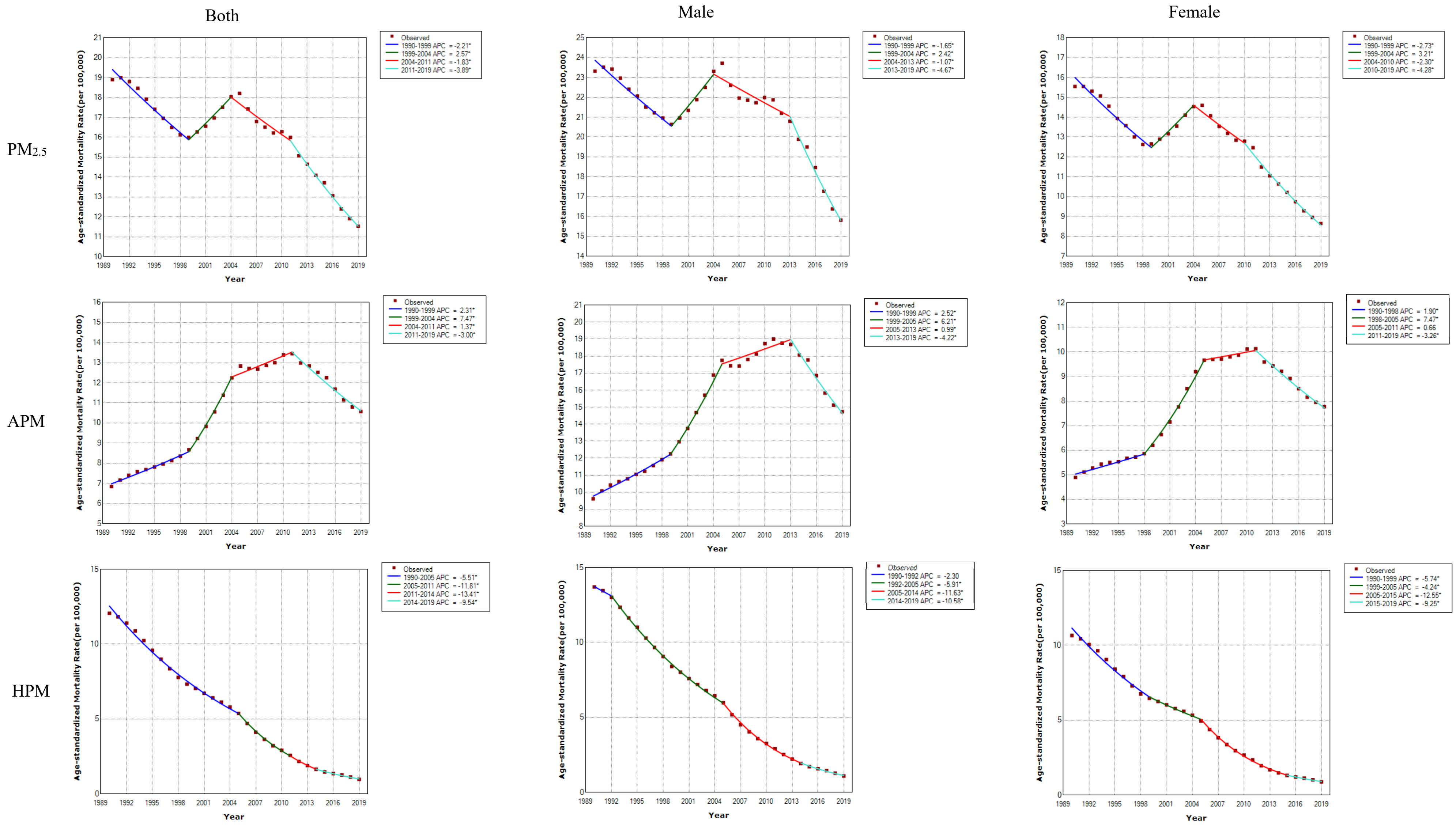

Figure S2. Joinpiont regression analysis of trends in the ASMR of IHD attributable to $PM_{2.5}$ (APM and HPM) in Jiangsu, 1990 to 2019.
*Indicates that Annual Percent Change (APC) is significantly different from zero at the alpha=0.5 level

Table S1. Mortality rate of IHD attributable to $PM_{2.5}$ by age, period, and median birth cohorts in Jiangsu, 1990 to 2019 (per 100,000)

Median period	Age group														Median birth cohort
	25-29	30-34	35-39	40-44	45-49	50-54	55-59	60-64	65-69	70-74	75-79	80-84	85-89	90-94	
$PM_{2.5}$														549.98	1900
													441.05	565.74	1905
												268.88	426.43	647.67	1910
											159.18	229.40	453.35	722.60	1915
										97.40	133.37	242.61	477.87	686.92	1920
									59.44	83.91	136.28	254.60	458.73	540.75	1925
								37.42	49.99	84.74	133.08	230.71	376.69		1930
							23.88	31.32	49.98	77.55	112.63	194.93			1935
						14.67	19.14	29.15	43.43	60.03	90.16				1940
					8.37	12.78	18.17	26.20	34.99	50.16					1945
				5.46	7.98	12.77	16.78	22.32	27.53						1950
			3.24	4.96	7.64	11.61	13.61	17.40							1955
		1.91	3.17	5.10	6.87	9.40	11.31								1960
	1.07	2.06	3.14	5.37	7.38	8.04									1965
1992	1.26	1.91	2.96	4.97	6.00										1970
1997	1.21	1.77	2.92	4.65											1975
2002	1.11	2.14	3.04												1980
2007	1.25	1.99													1985
2012	1.00														1990
2017															

Table S2. Mortality rate of IHD attributable to APM by age, period, and median birth cohorts in Jiangsu, 1990 to 2019 (per 100,000)

Median period	Age group														Median birth cohort
	25-29	30-34	35-39	40-44	45-49	50-54	55-59	60-64	65-69	70-74	75-79	80-84	85-89	90-94	
APM														203.76	1900
													167.82	265.44	1905
												103.72	205.04	392.10	1910
											62.38	112.31	279.33	535.45	1915
										39.08	65.99	151.21	358.64	581.28	1920
									24.20	42.04	85.65	191.60	392.55	481.97	1925
								15.51	25.41	53.50	100.71	197.76	339.17		1930
							9.95	16.15	31.83	58.69	96.87	175.80			1935
						6.13	9.96	18.70	33.05	51.70	81.48				1940
					3.50	6.64	11.80	20.11	30.29	45.40					1945
				2.30	4.09	8.25	12.91	19.34	24.96						1950
			1.35	2.55	4.87	8.90	11.79	15.78							1955
		0.80	1.64	3.30	5.32	8.18	10.29								1960
	0.44	1.04	2.03	4.16	6.43	7.32									1965
1992	0.62	1.21	2.29	4.32	5.46										1970
1997	0.76	1.37	2.77	4.24											1975
2002	0.86	1.86	2.54												1980
2007	1.09	1.82													1985
2012	0.91														1990
2017															

Table S3. Mortality rate of IHD attributable to HPM by age, period, and median birth cohorts in Jiangsu, 1990 to 2019 (per 100,000)

Median period	Age group														Median Birth Cohort
	25-29	30-34	35-39	40-44	45-49	50-54	55-59	60-64	65-69	70-74	75-79	80-84	85-89	90-94	
HPM														346.27	1900
													273.28	300.52	1905
												165.17	221.52	255.31	1910
											96.81	117.16	173.87	186.78	1915
										58.33	67.43	91.33	118.98	108.30	1920
									35.24	41.90	50.60	62.85	67.81	58.57	1925
								21.91	24.59	31.22	32.29	33.76	37.37		1930
							13.94	15.18	18.14	18.82	16.14	19.07			1935
						8.54	9.19	10.44	10.35	8.53	8.65				1940
					4.86	6.14	6.37	6.08	4.81	4.75					1945
				3.17	3.90	4.51	3.86	3.05	2.56						1950
			1.89	2.41	2.77	2.71	1.86	1.62							1955
		1.11	1.53	1.80	1.54	1.25	1.02								1960
	0.63	1.02	1.12	1.20	0.98	0.72									1965
1992	0.63	0.71	0.68	0.67	0.54										1970
1997	0.45	0.40	0.39	0.41											1975
2002	0.25	0.29	0.27												1980
2007	0.17	0.17													1985
2012	0.09														1990
2017															

Table S4. The wald χ^2 test of age, period and cohort effects

	PM$_{2.5}$ exposure			APM exposure			HPM exposure		
	χ^2	df	P-Value	χ^2	df	P-Value	χ^2	df	P-Value
**Both sexes**									
NetDrift = 0	410.72	1	<0.001	388.10	1	<0.001	7765.85	1	<0.001
All Age Deviations = 0	495.54	12	<0.001	595.40	12	<0.001	390.42	12	<0.001
All Period Deviations = 0	87.73	4	<0.001	435.93	4	<0.001	398.47	4	<0.001
All Cohort Deviations = 0	253.54	17	<0.001	293.50	17	<0.001	213.03	17	<0.001
All Period RR = 1	540.56	5	<0.001	674.04	5	<0.001	7847.85	5	<0.001
All Cohort RR = 1	1005.00	18	<0.001	869.34	18	<0.001	14927.06	18	<0.001
All Local Drifts = Net Drift	251.69	14	<0.001	290.67	14	<0.001	212.30	14	<0.001
**Male**									
NetDrift = 0	234.44	1	<0.001	357.67	1	<0.001	9446.05	1	<0.001
All Age Deviations = 0	889.24	12	<0.001	958.34	12	<0.001	691.42	12	<0.001
All Period Deviations = 0	45.43	4	<0.001	257.81	4	<0.001	342.18	4	<0.001
All Cohort Deviations = 0	359.99	17	<0.001	335.35	17	<0.001	342.89	17	<0.001
All Period RR = 1	302.25	5	<0.001	508.56	5	<0.001	9627.39	5	<0.001
All Cohort RR = 1	917.73	18	<0.001	637.87	18	<0.001	15444.51	18	<0.001
All Local Drifts = Net Drift	357.57	14	<0.001	332.25	14	<0.001	341.88	14	<0.001
**Female**									
NetDrift = 0	277.09	1	<0.001	104.05	1	<0.001	2346.26	1	<0.001
All Age Deviations = 0	183.65	12	<0.001	209.82	12	<0.001	128.00	12	<0.001
All Period Deviations = 0	71.78	4	<0.001	338.16	4	<0.001	208.18	4	<0.001
All Cohort Deviations = 0	80.45	17	<0.001	87.63	17	<0.001	56.86	17	<0.001
All Period RR = 1	372.40	5	<0.001	379.59	5	<0.001	2413.75	5	<0.001
All Cohort RR = 1	541.96	18	<0.001	503.80	18	<0.001	6189.46	18	<0.001
All Local Drifts = Net Drift	77.78	14	<0.001	84.21	14	<0.001	53.94	14	<0.001

Age-standardized mortality rate per 100,000 (95%*UI*)	Leading risks 1990	Leading risks 2019	Age-standardized mortality rate per 100,000 (95%*UI*)	Annualised rate of change(ARC), 2010–2019 (%，95%*UI*)
32.13 (22.58~44.23)	1 High systolic blood pressure	1 High systolic blood pressure	26.64 (18.21~36.56)	-0.65* (-1.04~-0.31)
25.43 (17.26~36.09)	2 High LDL cholesterol	2 High LDL cholesterol	20.33 (12.59~29.30)	-0.77* (-1.17~-0.53)
16.01 (13.35~19.65)	3 Tobacco	3 Tobacco	11.61 (9.19~14.75)	-1.11* (-1.50~-0.74)
12.54 (4.99~22.35)	4 Diet high in sodium	4 Ambient particulate matter pollution	10.57 (8.241~3.83)	1.50* (1.19~1.72)
12.06 (7.99~16.22)	5 Household air pollution from solid fuels	5 High fasting plasma glucose	8.98 (4.49~16.88)	-0.33 (-0.82~0.04)
10.28 (3.54~14.39)	6 Diet low in whole grains	6 Diet low in whole grains	7.89 (2.75~11.15)	-0.91* (-1.40~-0.60)
9.89 (5.14~17.96)	7 High fasting plasma glucose	7 Diet high in sodium	7.84 (2.53~15.60)	-1.62* (-1.98~-1.27)
8.29 (1.50~14.38)	8 Diet low in legumes	8 Impaired kidney function	6.17 (3.70~9.47)	-0.43* (-0.68~-0.18)
6.98 (4.15~10.21)	9 Impaired kidney function	9 Diet low in legumes	5.23 (0.66~9.29)	-1.59* (-1.96~-1.25)
6.84 (3.36~11.44)	10 Ambient particulate matter pollution	10 High body-mass index	5.21 (2.12~9.93)	1.70* (1.33~1.93)
6.38 (4.29~9.10)	11 Lead exposure	11 Low non-optimal temperatures	4.72 (2.87~6.89)	-1.00* (-2.21~-0.52)
6.30 (3.91~8.63)	12 Low non-optimal temperatures	12 Lead exposure	4.57 (2.87~6.95)	-1.16* (-1.54~-0.81)
5.10 (2.26~7.76)	13 Diet low in nuts and seeds	13 Diet high in trans fatty acids	3.02 (0.34~4.53)	-1.36* (-1.91~-0.96)
4.48 (0.40~6.39)	14 Diet high in trans fatty acids	14 Diet high in red meat	2.81 (0.42~5.51)	0.33 (-0.16~0.64)
4.33 (1.35~9.16)	15 Low physical activity	15 Diet low in seafood omega-3 fatty acids	2.48 (1.58~3.46)	-0.44* (-0.74~-0.17)
3.82 (1.45~5.96)	16 Diet low in fruits	16 Low physical activity	2.20 (0.53~5.46)	-2.34* (-2.88~-1.99)
3.33 (1.26~5.79)	17 Diet low in fibre	17 Diet low in polyunsaturated fatty acids	1.85 (0.26~4.05)	-1.94* (-2.25~-1.63)
3.25 (0.47~6.64)	18 Diet low in polyunsaturated fatty acids	18 Diet low in nuts and seeds	1.75 (0.58~3.18)	-3.70* (-3.82~-3.43)
3.18 (0.72~7.34)	19 High body-mass index	19 Diet low in fruits	1.68 (0.51~2.91)	-2.83* (-3.03~-2.60)
2.82 (1.08~4.04)	20 Diet low in seafood omega-3 fatty acids	20 Diet high in sugar-sweetened beverages	1.20 (0.67~1.83)	-2.25* (-2.56~-1.92)
2.73 (1.04~4.33)	21 Diet low in vegetables	21 Household air pollution from solid fuels	0.96 (0.33~2.16)	-8.72* (-8.86~-7.68)
2.56 (0.19~5.29)	22 Diet high in red meat	22 Diet low in fibre	0.87 (0.33~1.83)	-4.63* (-5.05~-4.16)
2.31 (1.51~3.23)	23 Diet high in sugar-sweetened beverages	23 Diet high in processed meat	0.43 (0.12~1.11)	0.21 (-0.18~0.39)
0.40 (0.19~0.82)	24 Diet high in processed meat	24 High non-optimal temperatures	0.22 (0.07~0.42)	-0.25 (-0.65~2.83)
0.24 (0.06~0.45)	25 High non-optimal temperatures	25 Diet low in vegetables	0.16 (0.10~0.34)	-9.75* (-9.86~-8.83)
-1.21 (-2.32~0.01)	26 Alcohol use	26 Alcohol use	-0.42 (-1.46~0.71)	3.77* (3.51~4.02）

Environmental and occupational risks
Behavioural risks
Metabolic risks

Figure S3. The rank of all risk factors for IHD in Jiangsu in 1990 and 2019.
